# Life after armed group involvement in Nepal: A clinical ethnography of psychological well-being of former “child soldiers” over time

**DOI:** 10.1177/1363461519850338

**Published:** 2019-06-13

**Authors:** Emilie Medeiros, Prabin Nanicha Shrestha, Himal Gaire, David M. R. Orr

**Affiliations:** 1 University College London; 2 Nepā School of Social Sciences and Humanities; 3 University of Sussex

**Keywords:** armed conflict, child soldiers, clinical ethnography, Nepal, resilience

## Abstract

Little is known about the longitudinal effects of early age involvement of young people in armed groups and their well-being as they return to strongly affected, politicised communities. Current research and policy are often driven by the assumption of a causal relationship between participation in this war experience and psychological damage. This article explores the role of young people’s armed group experience during the Nepal People’s War, compared with post-conflict stressors, in shaping intra-psychic impact and distress, and which processes enable well-being and resilient functioning. Findings are reported from an 18-month clinical ethnography of a cohort of 17 Nepalese young subjects, where participant-observation methods were used to explore their daily lives after exiting the armed group and follow-up research conducted six years later. The findings highlighted limited evidence for on-going intra-psychic impact and distress related directly to their armed group experience; when such distress occurred, it appeared to be generated more by the structural violence of their environments. The key constituents determining their well-being included: a sense of closeness through emotional connectedness with their family, ideological proximity with the values of the armed group, closeness in their bond with the community, and the social-emotional-economic capital available to them to navigate the harsh structural constraints of post-conflict life. These data further challenge the prevailing assumption that this war experience inevitably leads to psychological damage, and the article argues that structural violence often plays a predominant role in cases where psychological distress does arise.

## Introduction

The paradigm for research into the effects on young people of armed group participation during conflicts has undergone two significant shifts in recent years. First, a previously dominant psychopathology-centred approach, which overwhelmingly emphasised the identification of trauma symptoms within individual psyches, was challenged by psychosocial studies that expanded the scope of study to risk and protective factors within the social environment on return to civilian life (Amone-P’Olak, Lekhutlile, Meiser-Stedman, & Ovuga, 2014; Betancourt, Agnew-Blais, Gilman, Williams, & Ellis, 2010a; Betancourt et al., 2013; Fernando & Ferrari, 2013, Kohrt et al., 2010a; Tonheim, 2014). This shift clarified that young people uninvolved in armed groups were often affected by conflict in similarly pervasive ways, and required assumptions about the specific effects of participation to be revisited and differentiated more clearly from the generally applicable effects of structurally violent environments (Blattman & Annan, 2010; Eggerman & Panter-Brick, 2010). Second, a handful of researchers have questioned the interventionist assumptions inherent in the “child soldiers” concept underlying most studies and informing NGO and state/supra-state “Disarmament, Demobilisation & Reintegration” (DDR) programmes. These scholars noted how such assumptions foreground young people’s subjection to external forces and consequent trauma, portraying them almost exclusively as hapless victims of circumstances, in need of outside assistance. They critiqued the resultant disregard of young people’s capacity to respond positively to the adversity they have experienced and called for individual agency and social experiences to be given a more central role in analyses of young people’s resources and resilient functioning (Hart, 2006; Medeiros, 2012; Shepler, 2014). Research taking this approach started to question the assumed causal relationship between participation in armed conflict and psychological impact, often drawing attention to the influence of cultural context on how individuals responded and adapted (Blattman & Annan, 2010; Klasen et al., 2010; Medeiros, 2014a, 2014b).

This research has moved the field beyond static-cumulative models enumerating factors of vulnerability, to a more dynamic conceptualisation of bidirectional interactions between the individual and their environment, or ‘social ecology’ (Fernando & Ferrari, 2013; Kohrt et al., 2010a). This incorporates: the degree and nature of conflict experiences; indirect effects of violence through loss, insecurity, and displacement; socially-driven effects of stigma or acceptance, community cohesion, or breakdown; and availability of educational/employment opportunities (Adhikari et al., 2014; Betancourt et al., 2013; Miller, Omidian, Rasmussen, Yaqubi, & Daudzi, 2008). It also considers the processes of meaning-making in which young people engage during and after armed group experience (Denov, 2010; Kanagaratnam, Raundalen, & Asbjornsen, 2005; McAdam-Crisp, 2006; Medeiros, 2007). Domains found to underlie success in meaning-making include psychological competencies (sense of agency, empathy), access to cultural resources (spirituality, morality, rituals), social connectedness and quality of relationships with community and family, and commitment to a religious or political belief framework (Boothby, Crawford, & Halperin, 2006; Cortes & Buchanan, 2007; Medeiros, under review; Medeiros, 2014a).

This line of research has provided numerous insights, but surprisingly few studies have conducted long-term follow-up studies on the mechanisms that sustain or disrupt resilience as young people that were involved in armed groups transition into adulthood (Jordans et al., 2012).^1^ Such a shift in methods can produce new findings. For example, a retrospective study of youths in Uganda demonstrated that, over time, levels of well-being and impairment in daily functioning were similar between the young people who grew up in the war but did not participate with the armed groups, and those who did participate (Blattman and Annan, 2010). The only prospective longitudinal studies in the field, in Mozambique and Sierra Leone, identified that, with appropriate interventions, young people can adapt well to the challenges of reintegrating into society, and noted the importance of post-war experiences of discrimination in contributing to hostile behaviours (Boothby, 2006; Betancourt et al., 2010a). Here, it was only through long-term follow-up that it became possible to elicit the detail and nuance of the relevant social-emotional processes. Longitudinal understanding of the influence of armed group involvement remains at an early stage, with calls for more in-depth conceptualisation of the “dynamic developmental processes involved” (Yates, Egeland, & Sroufe, 2003, p. 244) over time (Betancourt, 2011).

The present qualitative study provides just such a longitudinal view of adjustment among a cohort of young people (now adults) formerly involved with an armed group in Nepal. Research with this population found links between armed group involvement and risk for depression, PTSD, and impaired psychological functioning (Kohrt et al., 2008), but also a diversity of experiences among young people participating in the armed group, with aspects of this affiliation sometimes conferring protective factors (Kohrt, Tol, Pettigrew, & Karki, 2010b; Kohrt et al., 2016). Studies have drawn attention not only to the role played by conflict experiences in young people’s long-term psychological functioning, but also to genetic (Kohrt et al., 2016), family (Kohrt et al., 2010a; Shakya, 2011), community (Kohrt et al., 2010b), and structural (Kohrt et al., 2012) influences on resilience. The current study uses observation and interview data to explore, over time, the role of their armed group experience compared with post-conflict stressors in shaping intra-psychic impact and distress, and what processes enable well-being and adaptive functioning. Though this study speaks to the aforementioned literature on trauma and resilience, the primary focus here is on the positive concept of well-being (Dodge, Daly, Huyton, & Sanders, 2012; Sen, 1993). A socially-rounded perspective on well-being recognises that it is multidimensional, incorporates both subjective satisfaction and the material conditions that enable humans to fulfil their capabilities,^2^ and allows for the influence cultural and community norms may have in shaping how they aspire to do so (Sen, 1993, 1999).

## Context

Over 16,000 people died in Nepal’s 10-year conflict between government forces and the insurgent armed group known in Nepali as *Māobādi*, the *party*, or the Maoist Communist Party of Nepal (CPN-M). The *Māobādi* insurgency challenged autocratic monarchic rule, its predominantly Hindu-based cosmology, and long-standing discriminatory governance that concentrated wealth, power, and development in Kathmandu, advocating instead for social and economic equality. Civilians were exposed to unpredictable violence by both parties (Pettigrew & Adhikari, 2010). Districts like Rolpa were targeted by brutal army operations indiscriminately targeting civilians assumed to be Maoist supporters (Onesto, 2005). This pushed many to join the Maoist ranks. UNICEF (2008) suggested that over 30% of *Māobādi* armed group members were aged between 9 and 16 years.

The “People’s War” started in the traditionally leftist region of Rolpa-Rukum in 1996, which remained its political stronghold and was popularly known as an area where “Everybody is a *Māobādi*!” Though central power remained largely secure in Kathmandu valley, the conflict spread to most of the country by 2001 and was particularly intense in remoter regions abandoned by central government (Thapa, 2003). There, the armed group operated as a parallel state with its own local government, justice, and infrastructure development bodies. A peace agreement ended the conflict in 2006. The *Māobādi* assumed a central role in Nepali mainstream politics, culminating in their election to lead the government in 2008, a few months before this ethnography started.^3^

## Methods

Kathmandu and Rolpa were selected as sites for this study because of their economic and developmental contrasts. Kathmandu is Nepal’s capital and political centre, while Rolpa represents “backwardness” in the Nepali collective imagination, due to its geographical remoteness, limited infrastructure, and poor communications. Rolpa also typifies structural violence, with limited access to health services or to economic and education opportunities, and widespread caste discrimination prior to the insurgency.

The study employed clinical ethnography (Calabrese, 2013; Herdt, 1999) of a cohort of young Nepalese actively involved in the Maoist armed group during the People’s War. Clinical ethnography is “culturally and clinically-informed self-reflective immersion in local worlds of suffering, healing and well-being” (Calabrese, 2013, p. 51). Employing standard ethnographic methods including participant observation and ethnographic interviewing, this approach also incorporates the application of disciplined clinical training such as assessment, formulation, and familiarity with therapeutic management of mild to severe forms of emotional suffering. This approach was necessary to ascertain with confidence the psychosocial functioning of these individuals and their emotional relationships, and was made possible through the fieldworkers’ therapeutic training and experience.^4^

Seventeen young *whole-timers* (10 male, seven female) participated in the study. *Whole-timers* lived fully “underground,” largely leaving behind their family and civilian identity when they enrolled for fear of government reprisals against them and their relatives. To avoid being caught, they were constantly on the move, usually at night. Although this group had occupied differing positions and ranks among the *Māobādi*, including cultural dancers, frontline reporters, and fighters, they were all exposed to extreme stress and violent activities during their affiliation. Their period as *whole-timers* had lasted between seven months and seven years, while they were between 10 and 16 years old. They were aged between 15 and 23 years on initially engaging with the ethnography in 2008, and between 23 and 31 years during follow-up in 2016.

Selection of participants aimed to capture a variety of environments and thus experiences during and after the conflict. Therefore, the cohort was recruited at two sites: the rural, *Māobādi*-supporting environment of Rolpa (*n* = 8) and the urban capital, Kathmandu, where the majority professed support for the traditional Hindu-based system and hostility towards the *party* (*n* = 9). All informants selected in Rolpa were from that region; all those recruited in Kathmandu were originally from outside the capital and most were only temporarily based there, usually to further their education. Recruitment through official NGO, CAAFAG (Children Associated with Armed Forces and Armed Groups), channels identified few *whole-timers*, so the snowballing approach was used, identifying cases through informants’ peers, community leaders, or mediators who knew about community members’ involvement during the war. Two young people declined to participate in the research.

The study gathered data through qualitative methods, allowing naturalistic disclosure of experiences. Participant-observation methods were employed. The first two authors lived in the informants’ homes for over 18 months (2008–2010), staying at each person’s home for a few days, depending on the circumstances and movements of the informant,^5^ before moving to the next. Formal interviews and innumerable informal discussions^6^ were held with the 17 key informants and most of their families, as well as secondary informants, including community members and key stakeholders. Follow-up ethnography was conducted with the same cohort in early 2016 for six weeks, six years after the first ethnography ended (10 years after the conflict ended). Fourteen of the original 17 informants were traced to their current locations, in Nepal, India, and Kuwait. Similar methods were used, mostly participant-observation and ethnographic interviewing. Facebook audio-call was used with the two informants based in the Middle East, but the researcher stayed with and engaged their families in their home of origin.^7^

Formal and informal mental health resources available where informants resided were mapped at the outset, facilitating referral for support where judged necessary. Several informants were referred to local organisations providing psychosocial support, mostly for the social and economic challenges they faced. During the follow-up phase (2016), however, mental health support was discovered to be close to non-existent in the areas some informants had migrated to, particularly as the *party* networks had often faded over time.^8^

Inductive data analysis began during participant-observation and led to an iterative process, as initial tentative thematic formulations revealed areas where deeper enquiry was needed. At later stages of the clinical ethnography, as patterns seemed to be emerging in relation to informants’ understandings of well- or ill-being and their constituents, emergent interpretations were put to informants to comment on, verify, or refute. At this point, the deductive framework provided by the authors’ clinical training in trauma and attachment theory was introduced and the data scrutinised for correspondences and contradictions with it. Data analysis of transcripts and field-notes took place in five stages, which were iterative rather than linear: (1) initial reading, impression formation, and note-taking; (2) assigning codes to individual units of meaning within the data; (3) collating codes into a smaller number of over-arching themes for potential use; (4) review and refinement of themes in the light of the clinical deductive framework; (5) further refinement of themes into the encompassing themes presented in this article.^9^

The project was approved by the Ethics Committee of UCL (application 1276/001). Institutional permission and support were obtained in Nepal from Tribhuvan University (Kathmandu) and CNAS (Centre for Nepal and Asian Studies), and with NEPA (School of Social Sciences and Humanities) during the follow-up.^10^

## Results

This section first explores findings on the post-conflict emotional lives of participants. Brief biographical accounts of two participants, here called Himal and Pabitra, are presented to provide context. The section “Overall psychological functioning post-armed group participation” then reports the findings, which indicate that armed group participation per se did not influence psychological well-being in deterministic fashion within this group. Two themes are then discussed which describe alternative processes important in shaping the well-being of these and other young people over time: “Inter-subjective influences” and “Structural influences.”

Both Himal and Pabitra are from Rolpa and their post-conflict experiences illustrate particularly clearly the socio-emotional processes that influenced well-being across the wider group of participants, both those recruited in Rolpa and in Kathmandu. The young people who enrolled among the *Māobādi* were predominantly from remote areas comparable to Rolpa in respect of the social and structural constraints analysed here. For all the value to be found in detailed case presentations, no implication is intended that Himal and Pabitra’s stories are at all archetypal; other participants’ life trajectories distinctively differed.

### Brief biographies

#### Himal

Himal, of Kham Magar ethnicity,^11^ lived in a remote village of Western Rolpa located a two-hour steep walk from the closest town. He was 20 in 2008 (27 at follow-up). In 2008, he was living in his parents’ home with his parents, two younger siblings, his wife Laxmi, and their two children. The family was very poor, their land being in the least fertile side of the village; animal farming produced barely enough for them to survive for seven months of the year. During winter, the more able members of the household often had to work in exploitative conditions in the Kathmandu brick factory to boost their income. Himal left in 2013 to work in Kuwait as an external painter, to maintain the family’s standard of living.

Himal attended school until Class 8, despite constant police harassment in his village and school during the insurgency. The armed forces deployed intrusive searches and intimidation strategies towards local teenagers, who were all assumed to be *Māobādi* supporters, a crime punishable by death. Most young villagers would therefore often sleep in the bush at night to protect themselves, regardless of their allegiance. He described going underground at barely 14 years old, in response to this pressure, hoping for the infrastructural development advocated by the armed group: “Just for a road [to my village] ….” Himal was a member of the PLA (People’s Liberation Army) when he met and married Laxmi, a *party* commander. She proudly described her gratitude to the *party* for promoting her to this status over others who, unlike her, had been sent to school. Himal fought in the battlefield for three years until he got injured and Laxmi became too pregnant to continue living on the run.

#### Pabitra

Pabitra, of Magar ethnicity, was 18 at contact (25 at follow-up). Her village, although near Himal’s, is more accessible, and enjoys more fertile lands for agriculture, better water, and better health facilities. Their fields and animals provided the family with a reasonable income. Pabitra’s father died when she was one year old, so her siblings mostly raised her. She suffered a foot deformity resulting from a domestic injury, but never considered herself “disabled.”

Pabitra stopped school in grade 6 due to unbearable police threats during searches for *Māobādi*. Government forces eventually placed her under a probation order, putting her at risk of “disappearing” from the police station, like many others in the area. This prompted her to join the *party* at age 13. She was in charge of women’s social and health awareness, a role that suited her values and was convenient given her physical challenges. When the war ended three years later, she left the armed group with her commander’s permission to return to civilian life. Later she married Prabin, who had been a PLA fighter with the *party* from the age of 14 and whom she had gotten to know while they were both underground during the war. Frustrated with the lack of prospects with the United Nations-led DDR cantonments,^12^ Prabin left the *party* to work in Qatar for a few years, before settling again with Pabitra and their two children.

### Overall psychological functioning post-armed group participation

When prompted or interviewed about their emotional worlds, most informants did not share any current psychological difficulty related to their experience or exposure to violence when underground. As Himal, 20, explained:

I used to feel enraged about [police threats in the schools] and that helped to give me the courage to fight in the PLA. (…) Even in the PLA, I used to have nightmares about that, that they will finish us. (…) After I left I sometimes thought about it and had these nightmares, especially as my best friend was killed just next to me … but these days I don’t think about it that much and I don’t have these dreams any longer. (2008–2010)^13^

Like Himal and others, Pabitra did not disclose any psychological impact as such about her experience or any significant anxieties or distress in the conversations or formal interviews with her. In fact, she eventually became exasperated with questions about this: “But *didi* [respectful title to the researcher], I really don’t have any *chinta* [anxieties] or problems, really!!!” (2008–2010). The informants’ statements about their subjective worlds were confirmed when regularly triangulated with the family members or close friends who would have intimate knowledge of the young people. As the researchers’ on-going visits built up trust, participants usually disclosed anxieties or any difficult feelings or memories. There were few cases where the informants shared with the ethnographer that they suffered from traumatic impact. One of these was Nona, 18, who only agreed to participate in the ethnography if the researcher did not ask about her experience in the *party* in order to avoid bringing the scary memories back. Her mother and her sister had, with Nona’s consent, shared their concerns about her behaviours and changes in her personality after her return.

In their daily lives and across multiple social contexts, many informants’ expressions and psychological positions indicated positive adaptation overall. Signs of psychological distress were often absent and no particular emotional reactions were observed in response to triggers associated with their armed group experience (e.g., related political programmes in the community or on radio; related discussions with neighbours, family, or friends). Some did react to particular war experiences that went beyond the experience of armed group involvement and exposure to conflict per se: for example, Kum and Krishna presented some post-traumatic signs that seem to stem from their 18 month experience of torture when detained at the age of 14, following their arrest for PLA membership by government forces. Others presented some psychological difficulties as a result of domestic violence and attachment issues or issues of neglect. Three of the informants came from emotionally and often physically abusive families, where parents had died and/or the family had mostly abandoned them before their involvement in the armed group. Their domestic situation did not change when they returned and this continued to affect them at follow-up. Geheraj (aged 15 in 2008), one of the few informants whose battlefield exposure clearly and directly resulted in significant impact, was one of these three and later experienced full psychotic breakdown that led to his hospitalisation. These observations point to some of the factors that determined long-term well-being to a greater extent rather than participation in conflict in itself, explored in the next section.

### Inter-subjective determinants of well-being

Family and community relationships that enabled a sense of social-emotional closeness and of value consonance with a reference group were crucial determinants of these young people’s well-being. Intimacy, emotional safety, validation, congruence and continuity of values, and belonging were of great value in the post-insurgency landscape, helping to embed them within close social circles on their return from the armed group. These are described under the sub-themes “Emotional proximity,” “Ideological proximity,” and “Closeness with community.”

#### Emotional proximity

Both observation and participants’ own comments revealed that feelings of closeness with their intimate circles such as family and partners were a crucial determinant of their well-being over time. The quality of attachment between informants and their close family members before, during, and after their involvement in the armed group was instrumental in several ways. Its presence or absence shaped the quality and formation of love partnerships as well as how they emotionally invested in the Maoist group itself, the social relationships they were capable of forming during and after their experience, and the socio-emotional resources they possessed to help them cope with the challenges they faced during adulthood.

Quality of attachment, manifesting in subjective emotional proximity to the caregiving figures to whom these young people returned from their life “underground,” was crucial in enabling them to feel loved, cared for, and protected, which in turn shaped their overall sense of emotional safety in building relationships with others. Pabitra, for example, was particularly close to her mother and siblings, who have had a very caring attitude towards her throughout her young life. Her family’s unusual living arrangement exemplifies this. All her sisters have chosen to live at their *maiti* (maternal family compound) with their children, instead of at their husband’s family home as culture prescribes. Pabitra, too, continued to reside in the maternal compound and look after her mother and her children, thereby transgressing patrilocal norms. She maintained contact with her family while she was “underground,” and they were supportive of her throughout. The experience of a safe attachment shaped her capacity to feel emotionally connected with her family, and therefore to use them as an emotional support upon her return. This resource also enabled her to form a sense of closeness with her peers and partner before, during, and after her armed group involvement. The contrast stood out starkly where attachment figures were absent, such as with Renuka, 15. Her father abandoned the family, her mother remarried,^14^ and both stopped contacting her. Her care was left to her aunts and grandparents. Perceiving Renuka’s care as a burden, they were either abusive or neglectful. Her family was unconcerned when she left them to join the party at age 10 or when she returned a few years later; when the *party* asked her to go back home at the end of the conflict, she experienced it as another abandonment, this time by her new family. No warmth or attention was observed during her interactions with her caregivers, from whom she frequently ran away.

Where informants’ parents experienced mental health problems, this affected their sense of emotional proximity and therefore sense of safety, sometimes hindering their capacity to cope with adversity. For example, Himal’s father had been chronically intoxicated since retiring from the Indian army. His speech was incoherent even when sober and he was incapable of contributing labour towards household survival. It was clear in Himal’s language and posture that his father’s alcoholism was a great burden, for which he constantly expressed embarrassment. As the eldest child and son, Himal early on had to become the “breadwinner” and the main provider of care for his siblings, contributing to his sense of insecurity.

Perceived emotional proximity with the love partner played an important role in the well-being of many of the young people. Continuity with their partner’s values provided them with a sense of familiarity and emotional support and its absence led to observable distress. Himal and Laxmi had a love marriage sanctioned by their commander while underground. They described sharing similar ideas (“*bichar milne*”), political activism, war experiences as PLA fighters, and cultural values stemming from shared caste background and shared identity as hard-working farmers. They were initially observed to have a very close relationship, caring for and supporting each other in their dedication to finding means to educate their children and offer them better life opportunities: “We often sit together … Sometimes we remember the time when we were involved [with *Māobādi*] and when we were in the battlefield together” (2008–2010). In the first years after leaving the armed group, informants sometimes found it complex to sustain the *Māobādi* belief system in their daily practice. Key to their well-being was a sense of proximity and emotional safety with their partner that eased this negotiation through shared core beliefs and values. The follow-up research uncovered the negative impact on Himal when this emotional resource was no longer available to him. After living apart for three consecutive years with limited exchanges, Laxmi tearfully explained:

I worry a lot about Himal. I’m no longer able to repay the loan because he does not send the money and I think he spends his earnings now on alcohol. He doesn’t often call these days and he doesn’t talk to me any longer about what’s happening with him or what bothers his *man* [heart-mind]. (…) When he calls, he is often drunk and he has sometimes said we should continue life without him. (2016)

#### Ideological proximity

The young people’s psychological health was mediated by their identification with the belief system of the *Māobādi* group, which mutually validated their own values, those of their referent groups, and their previous participation in conflict. Congruence between their families’ values and those of the *Māobādi* contributed to closeness, emotional safety, and cognitive consonance as armed group members and on their return. This was significant for many informants, regardless of their family’s active allegiance to the *party*. Pabitra explained:

I, my older sister and my two older brothers joined the *party*. After joining the party, we didn’t meet a lot of people from outside [the *party*], that’s why also our marriage happened with the people from within. Then again I am not much interested in people from outside. (…) The people of the party are different from the people of the village. The people from the village drink, play cards, gamble. If they like any girl, they bring her/marry her even if there is a wife at home. But the people from the party have been staying in discipline, they don’t drink, don’t gamble or do multiple marriages. That’s why I like people from the *party*. (2008–2010)

After her father died, Pabitra’s family had lost faith and gradually discarded many traditional beliefs and practices, such as expensive grieving rituals or gender expectations. This resonated with some crucial tenets of the *Māobādi* agenda for societal reform (to abolish religious, caste-based, and supernatural practices). Many of our informants’ families, particularly in Rolpa, shared many *party* values. We found that where there was less values congruence, young people navigated less smoothly their re-entrance into civilian and family life. Gender issues came into play in more orthodox, Hindu-dominant communities that preserved a different image of women than did the *party*. Confronting the challenges of returning to those communities was difficult for young women (Hutt, 2012).

The young people’s adherence to some of the tenets of the *Māobādi*’s worldview was an important determinant of their well-being and varied greatly between participants. Pabitra exemplifies congruence with the *party*’s ideological doctrine in rejecting supernatural beliefs and professing gender equality and rights. Prior to joining the *party*, she was the first woman in her area to plough the fields, transgressing the traditional belief that to do so risked illness or infertility. Pabitra also spoke of her “heightened *chetanā* [consciousness],” which resulted in newly gained social status in her community. Indeed, affiliation to the *party* provided an opportunity for youths to be heard and recognised as adults or a *Thulomanche* (big man). Fear or respect for informants’ armed group links conferred on them a certain authority in their community. In her village, Pabitra has confidence in her ideas and often denounces people who contravene moral principles, regardless of their gender or status. She is also unusually autonomous and ambitious. She set up her own tailoring shop and argued for her financial independence within married life.

Another crucial mediator of well-being was whether the former armed group validated the young people’s experiences with them. This recognition only occurred with a third of the informants, usually when they had remained *whole-timers* and actively involved with the *party* up to its election to government. Himal’s narratives of despair evidence the importance of acknowledgment of their sacrifices. He was seriously injured in the battlefield and sent for treatment at home. Subsequently, nobody from the *party* checked on his health, offered to cover his treatment fees, or enquired about his return. For the several informants who had remained *party* loyalists and had sacrificed much without recognition from the *party* in the 2008–2009 period of political glory, debate in 2015–2016 about access to the DDR reintegration package provoked renewed bitterness and anger. This was the case for Himal and his wife who, as long-standing former PLA members, were entitled to access the package. Their former commander did not support their application, which, in the context of their financial marasmus, would have repaired the bitter relationship. The *party*’s inability to validate their contribution to the insurgency disillusioned Himal, whose emotions were characterised by recurring anger, sadness, and rumination about betrayal and abandonment.

#### Closeness with community

A sense of closeness with their community was crucial when their involvement with the armed group appeared to be trivialised in this way, as it enabled them to access informal emotional support. Involvement in insurgency was a normalised and often praised experience for many in Rolpa’s social fabric, as Pabitra explained:

When I came home after leaving *the party*, I didn’t have to face any kind of problem or challenge. Nobody made any negative statement to me, I didn’t have to see any alcohol consumption or drunk person. The people from the village neither said good or bad that I went there … then again, all the people are from the *party*. (…) All have been *Māobādi* since the beginning! (2008–2010)

The war experience among youths in this area became a source of social and professional capital. This positive social value provided them with purpose, status, pride, group identity, and belongingness through shared experience. “Love of the people” can be found in Maoist memoirs as a core underpinning to narratives of enrolling for a life underground (Hutt, 2012), and indeed the *party* continued to be socially active and somewhat effective in addressing community concerns within the Rolpali post-insurgency landscape. Social support for the integration of former party members was facilitated by *party* activities or structures that aimed at “preserving social harmony,” such as the YCL (Young Communist League). For instance, Himal was initially a YCL member engaged in settling village-level disputes, which provided him a role and certain authority as a former armed group member.

In addition to normalised participation in the armed group, a crucial determinant in shaping young people’s emotional health was access to collective support mechanisms. These mechanisms consolidated their sense of belonging and collective identity resulting from shared suffering. Many informants from Rolpa accessed these supports, but in Kathmandu, such targeted collective support mechanisms were largely unavailable. Pabitra’s brother and brother-in-law lost their lives whilst fighting in the war and her grief for these martyrs (*shahid*) was shared with many neighbours and peers, giving rise to shared sorrows, sympathy, and mutual emotional support. During the first years after the insurgency, the *Māobādi* group operated as a sub-culture, in the sense that it remained apart from Nepal’s dominant, primarily Hindu-derived cosmologies, and yet, as the Maoist efforts to abolish this hegemonic framework had been unsuccessful, the group had to recognise and co-exist with its structures and practices. This reality was aptly captured by Deepak, 20, a Dalit (“untouchable” caste), when he observed with a cynical smile that the *Māobādi* were now (2009) the “fifth caste.” His comment framed the *party* within the overarching cosmology that traditionally identifies four Hindu castes. While set outside and apart from those who identify more closely with Hindu values by virtue of forming a distinct, self-contained, fifth group with its own cultural tenets, the *party* was unable to break away as fully from the Hindu schemas of inequality as they had hoped. The *party* also set up social groups like the YCL, *Jaljhala* radio (a *party*-funded Rolpali station), the Martyrs and Injured Association, and regular cultural programmes, providing their former underground members with a platform for continued political activism, a sense of purpose, and informal emotional support. Significantly, the few female informants from religiously and politically orthodox communities (Hindu, who practise caste discrimination and are politically unsupportive of the *Māobādi*) and/or marginalised families had a different experience. They did not access this informal support, either because it was not available in their areas or they did not identify with this collective experience, depriving them of potential coping resources. In the “heartland of the Maoists,” by contrast, Rolpalis benefited from maintaining these socio-emotional bonds and reinforcing their shared experience; even outside their home environments, Himal and fellow migrants in Kuwait were able to draw on these bonds to fortify each other in the face of prolonged family separation, financial pressures, and harsh working conditions.

### Structural influences on well-being

Post-insurgency, young Rolpalis were faced with structural violence that remained as deep as ever, with limited opportunities and infrastructures, compounding their losses. Informants’ predominant concerns centred more on surviving within this challenging environment than on their conflict experiences. For a majority of the sample, this shaped significant existential anxiety blended with a feeling of helplessness or low mood, often referred to by the idiom *dikka*. The term *dikka* is commonly used in Nepali and conveys varying mixtures of boredom, fatigue, irritation, upset, worry, and day-to-day frustration. For this cohort in Rolpa, however, *dikka* emerged in reaction to being trapped in an environment of hopelessness, where the ways out seemed close to “enslavement,” regardless of the trajectory chosen:

People experience *dikka* when there are no facilities, no electricity in the village, no opportunities … It is difficult and people don’t feel like working and going to the fields. (…) I started to feel it when I returned from the *Māobādi*. I feel it every time when I am at home so then I just feel I should go abroad to earn money. (Himal, 2008–2010)

Thus, the third crucial process affecting these young people’s psychological well-being was their ability to navigate this landscape by accessing the various forms of capital needed to fulfil their social duties and responsibilities.

Access to social-economic capital via their families was central in determining their trajectories. Himal’s family’s inability to provide him with initial social and economic capital affected the quality of opportunities available to him during migration. The interest rates he negotiated for his loan were extortionate and the broker he found was unreliable, so the work positions he could access were limited and risky. As an exterior painter-decorator in the extreme weather conditions of Kuwait, he struggled to perform his work and was barely able to repay his loan interest several years after migrating. This was a common challenge among the third of study participants who had migrated between 2008 and 2016. Thus, Himal’s efforts to overcome his family’s poverty were largely frustrated.

Maintaining association with the Maoist group provided some informants with significant socio-professional capital, enabling them to navigate the structural constraints of their environment and contributing to their emotional well-being. For example, Pabitra’s strong relationship with the *party* and her family’s political capital allowed her to receive foot surgery via the *party*-led Martyrs and Injured Association and to be given priority in the CAAFAG programme to access tailoring training and a sewing machine. These opportunities, coupled with social networks she built before and while underground, allowed her to set up a successful local tailoring business, granting her further independence before committing to marriage and parenthood.

The capital informants accessed through their *party* involvement further strengthened their well-being by validating the legitimacy of their involvement in the war. This capital was derived indirectly from the *ijjat* (social prestige) associated with the materialisation of some social-economic reform in the region,^15^ and directly from the individual benefits derived from links with the *party*. The young people felt that their previous actions, sorrows, and sacrifices were worthy. Bhawana, 25, comments:

[Involvement] is *balidān* [sacrifice] indeed, like the example of Ram. He became a *shahid* [martyr] in the battle. He sacrificed his life there, but I couldn't make such a sacrifice. (…) If you sacrifice, your popularity increases. Even if there is sorrow in the family, everybody in the society and community recognises you as the daughter of Mr or Mrs who sacrificed for the country … I was popular in the society until I stopped working for the *party*. Now that work doesn't have any meaning or value. But if I had been able to sacrifice, everybody would have respected me now. (2008–2010)

Furthermore, accessing capital was also crucial to enabling young people to perform the gendered social duties expected of them. While Himal, his partner, and his mother could all work seasonally in brick factories, only Himal could migrate for labour to the Gulf. This gendered trajectory seemed to offer the family a chance for escape from their financial straits:

I think it was good that I got involved at this age [13], there is a time in your life for everything. Before, you just don’t have any duties and you just look after yourself. If you become a martyr for your country then it’s important and your family will cry but it will not impact them in the same way. Nowadays, I am in charge of my family so I couldn’t join politics. (2008–2010)

Sustaining his masculinity involved performing his duties as elder son and brother, husband and father, which was a central concern for Himal when he returned to the community. However, this sacrifice led him to despair. Deprived of the emotional support of his family and faced with harsh, exploitative work conditions in Kuwait, during the follow-up phase Himal was drinking to cope. Trapped in a vicious cycle where he could not return home until he repaid his high-interest loans, let alone accrued his own savings, he drank more to cope. Consequently, he spent the little money he earned on alcohol without ever being able to repay his debts. He described occasionally contemplating suicide. Other study participants during follow-up described similar migration experiences of dilemmas as family breadwinners, entanglement with abusive work schemes, and spiralling family loans.

## Discussion

These findings shed light on the balance between the direct effects of participation in the *Māobādi* armed insurgency and wider influences, including the crucial role played by structural stressors, on the well-being of a group of Nepali young people. Qualitative data obtained during continued engagement with their daily lives brings into question prominent research and policy assumptions that the psychological impact of armed group involvement per se is necessarily the dominant determining factor in well-being outcomes in this population. The data pointed to the significance, among the mechanisms underlying their psychological functioning, of the intimate environment and structural stressors over trauma directly linked to participation with armed groups. On-going structural violence appeared more significant in affecting this group’s psychological functioning than the war experience itself. This fundamentally challenges research that continues to place the intra-psychic impact of armed group participation at the core of analysis of the subjective worlds of this population (Amone-P’Olak et al., 2014; Bayer, Klasen, & Adam, 2007; Bissouma, TeBonle, Yeo-Tenena, Moke, & Kpre-Koiho, 2010; Okello, Onen, & Misisi, 2007; Ovuga, Thomas, & Moros, 2008; Pfeifer and Elbert, 2011). Instead, it suggests that the social and structural environment should be placed at the heart of the study of young people’s subjectivity. Interestingly, our findings were similar to those of Eggerman and Panter-Brick (2010), who studied young people affected by political violence in Afghanistan, and of Kohrt et al. (2012), who found depression among Nepalese adults to be affected more by structural marginalisation than by exposure to violence during the People’s War (most participants were not directly involved with armed groups in either of these studies). The outcomes described in our study converge with the scholarly trend to recognise the dialectical relationship between the social, political, economic, and emotional determinants of resilience in the subjective worlds of young people living in structurally violent environments (Blattman and Annan, 2010; Medeiros, 2007, 2014b; Newnham, Pearson, Stein, & Betancourt, 2015).

A further contribution of this study is to identify the significance of the distinctive socio-political values that held sway in much of Rolpa. Many studies of the consequences for well-being of young people’s armed group involvement have emphasised the negative effects of stigma and marginalisation, both in Nepal (Kohrt et al., 2008, 2010a; Shakya, 2011) and elsewhere (Annan, Moriah, & Aryemo, 2009; Betancourt et al., 2010a). This ethnography offers a contrasting example of an environment that normalised and even lauded engagement with the armed group, and the role this played in participants’ well-being. Pro-armed group sentiment in the community was instrumental in maintaining consonance and a smoother meaning-making process of their war experience. DDR responses to “child soldiering” are often informed by interventionist preconceptions that conceive these subjects as passive victims of their environments, whose traumatic suffering stems primarily from their negative psychological experience in the armed group, and whose lack of personal resources prevents them from overcoming adversity without psychological support.^16^ This study challenges the side-lining of cultural context inherent in these assumptions, reinforcing instead the importance of local socio-political norms and cultural values in understanding the dynamics of psychological health among returning young people. Beyond this specific point, this study’s in-depth focus on the complexity of young activist subjectivities further challenges the assumptions of victimhood underlying discourses of “child soldiers.” Among this group, detectable psychological impact and distress stemming directly from the armed group experience were limited.^17^ The Rolpa findings thus contribute to scholarship that challenges preconceptions about morbidity and re-focuses research on the possibility that positive growth or protective influences might also emerge from young people’s war experiences (Hart, 2006; Klasen et al., 2010; Punamäki, 1996; West, 2000). Anthropologists have criticised the export of “Western” understandings of trauma through its effects within the individual psyche, which universalises a potentially inappropriate pathologising perspective (Fassin and Rechtman, 2009; Summerfield, 1999); the findings emerging from this study too support a more culturally-informed framing of subjectivity that moves beyond an overriding focus on individual psychology.

The unique access to subtleties of everyday presentation and deeply personal narrative disclosures that enabled these findings was achieved through clinical ethnography with a longitudinal approach, living with young informants and their intimate social circles for sustained periods of time. The original use of participant-observation methods with this group allows exploration of how psychological well-being intertwined with the constraints and possibilities of the cultural milieu, as these young people transitioned into adulthood. Involvement over an eight-year period with these young people’s worlds provided exceptional access to the complex dynamics of the social, emotional, and political processes underlying their coping mechanisms and experiences of suffering upon return to their communities (Medeiros, under review). Such involvement enabled the researchers to build on existing studies that rely on static descriptions of risk factors, to offer a more dynamic and in-depth analysis of subjective worlds, made possible through the original use of both clinical and anthropological methods. This study thus makes a distinctive contribution in helping to advance the shift towards incorporating social ecologies into the psychological understanding of this population and developing longitudinal studies (Betancourt et al., 2010b; Boothby, 2006). It highlights the benefits of this innovative methodological approach for use in this and other post-conflict settings, to investigate the long-term effects of comparable life experiences within their particular social ecologies.

The study has limitations, which means that our findings should not be overstated. The sample was small, due to the in-depth clinical ethnography practised, and cannot be considered generally representative of youth experience in Rolpa, much less beyond. Its contribution lies in exploring pathways and influences that impact these young people in different ways, rather than in making sweeping generalisations about contributing factors or outcomes. Additionally, while the close relationships developed over time by the researchers enabled unique opportunities for disclosures and observations, they doubtless also situated us within the communities and at times may have paradoxically made community members reluctant to inform us of things that outsiders—less involved in the community—might be more readily told. Nevertheless, these limitations do not negate the implications of the outlined findings for policy and practice.

## Conclusion

This research challenges preconceptions of victimhood in demonstrating that severe psychological impact and distress are not universally an inevitable consequence of participation in armed conflicts. It argued further that structural violence often plays an instrumental role in cases where psychological distress does arise, thus calling for integrating the multiple layers of psychological and social influences that affect young people’s successful adjustment in the aftermath of armed group involvement. The approach advocated requires a shift in the premises used by policy-makers and service designers. Their understanding of the needs of young people involved in armed groups needs to be realigned with the lived experience of the social-psychological processes identified in this and related bodies of research to ensure that their psychological well-being is preserved and their reintegration to civilian life is successful.

## Notes

1 See Betancourt, Brennan, Rubin-Smith, Fitzmaurice, and Gilman (2010b) and Boothby et al. (2006) for exceptions.

2 In Sen’s terms, this fulfilment constitutes their “functioning.”

3 Some Rolpalese leaders, such as Barshaman Pun and Ownsari Gharti Magar, reached the forefront of the political scene.

4 Author 1 (Medeiros) is trained as a medical anthropologist and clinical psychologist, and has significant experience of clinical assessment and engagement in the psychological recovery of young people in several conflict and non-conflict affected environments. The Nepalese research assistants (authors 2 and 3: Shrestha, Gaire) have professional backgrounds in counselling and social (psychosocial) work, enabling all three fieldworkers to draw on culturally-informed clinical judgment in assessing the psychological and social well-being and functioning of participants, mediating the relationship, and eliciting willingness to confide intimate experiences. Author 3 was involved continuously throughout the initial ethnography and follow-up; Author 2 became involved at the follow-up phase.

5 The informants were very mobile, especially during the 2008–2010 ethnography, since they were at a transitional phase in their lives, and many had not yet settled socially or economically.

6 Informal interviews were conducted whenever the opportunity arose in informal conversations or in the context of a specific subject emerging in the interaction, and could range from a few minutes to over an hour, making it difficult to quantify them precisely.

7 The data were not audio-recorded, nor were notes generally taken during the exchanges, both to maintain free-flowing rapport and because people were very wary of being recorded in the post-conflict situation. The interviews were transcribed as accurately as possible immediately afterwards, relying on the memories of both researchers involved and on occasional notes taken. The full accuracy offered by recording was therefore sacrificed to allow informants to speak more freely. Interviews were conducted mostly in Nepali and occasionally in Kham Magar, and led by the lead researcher with interpretive assistance and input from the research assistants. All interview transcripts were translated by a Nepali professional with expertise in translation.

8 This posed ethical challenges to the team, particularly for informants away from their usual support structure who were reluctant to share their sorrow with their loved ones. In those instances, when risks were identified, they were mitigated through informal follow-up by the research team to determine the severity and offer what ad hoc support could be provided, e.g., supporting one participant who had migrated to the Gulf to locate an advocate.

9 Authors 1 and 3 worked collaboratively on stages 1–4 during the initial ethnography, and both worked closely with author 2 on the same stages during the follow-up phase in 2016. Moreover, author 1 provided overall direction for the analytic process, established the deductive framework, led the clinical interpretation, and undertook stage 5, while the research assistants led the interpretation of cultural concepts, interpretation between Nepali and English (and at times Kham Magar), and fed back their sense of what transference occurred in the relationships with participants. Author 4 was not present in Nepal and did not directly undertake data analysis. His contribution lay in conceptualisation of the article, theoretical interpretation, integration with the literature, and co-writing the article.

10 Author 1 was affiliated to the CNAS throughout the research and a formal collaboration was set up for the follow-up research in 2016 between UCL and the academic organisation NEPĀ, where authors 2 and 3 were affiliated.

11 The Kham Magar are of Tibeto-Burman origin and mostly populate the Middle Hills of mid-western Nepal (Rukum-Rolpa region). They constitute the third biggest ethnic group (*janajāti*) in Nepal and were particularly prominent in constituting the forces of the *Māobādi* during the People’s War.

12 The DDR process aims to contribute to the security and stability of a conflict area by removing the arms of the fighters involved (Disarmament) and by dismantling the military structures through a transfer of those combatants into cantonment sites (Demobilisation), before supporting this population in the integration into civilian society (Reintegration).

13 The period of fieldwork during which these data were gathered is given in brackets at the end of each quote.

14 Traditionally, the mother cannot bring with her the children from her previous marriage, who are usually given to the paternal grandparents for care.

15 For example, the 2008 *Māobādi-*led government prioritised the funding of infrastructure and roads in remote areas of Rolpa, reducing isolation.

16 See policy interpretation of international instruments concerning CAAFAG in Nepal (Shamrock, 2012; Thomas, 2010).

17 This is in line with existing studies that sought to quantify the long-term incidence of mental health problems among former child combatants in comparison with non-combatant peers, and found that in some instances the differences were of limited significance (Blattman and Annan, 2010; Castelli, Locatelli, & Mark, 2005).
